# A Review of Computational Methods for Finding Non-Coding RNA Genes

**DOI:** 10.3390/genes7120113

**Published:** 2016-12-03

**Authors:** Qaisar Abbas, Syed Mansoor Raza, Azizuddin Ahmed Biyabani, Muhammad Arfan Jaffar

**Affiliations:** College of Computer and Information Sciences, Al Imam Mohammad Ibn Saud Islamic University (IMSIU), Riyadh 11432, Saudi Arabia; smraza@imamu.edu.sa (S.M.R.); aabiyabani@imamu.edu.sa (A.A.B.); arfan.jaffar@ccis.imamu.edu.sa (M.A.J.)

**Keywords:** gene, DNA, non-coding RNA, micro RNA, computational intelligence, support vector machine, Bayesian networks, genetic algorithm, neural network, deep learning

## Abstract

Finding non-coding RNA (ncRNA) genes has emerged over the past few years as a cutting-edge trend in bioinformatics. There are numerous computational intelligence (CI) challenges in the annotation and interpretation of ncRNAs because it requires a domain-related expert knowledge in CI techniques. Moreover, there are many classes predicted yet not experimentally verified by researchers. Recently, researchers have applied many CI methods to predict the classes of ncRNAs. However, the diverse CI approaches lack a definitive classification framework to take advantage of past studies. A few review papers have attempted to summarize CI approaches, but focused on the particular methodological viewpoints. Accordingly, in this article, we summarize in greater detail than previously available, the CI techniques for finding ncRNAs genes. We differentiate from the existing bodies of research and discuss concisely the technical merits of various techniques. Lastly, we review the limitations of ncRNA gene-finding CI methods with a point-of-view towards the development of new computational tools.

## 1. Introduction

**Figure 1:**
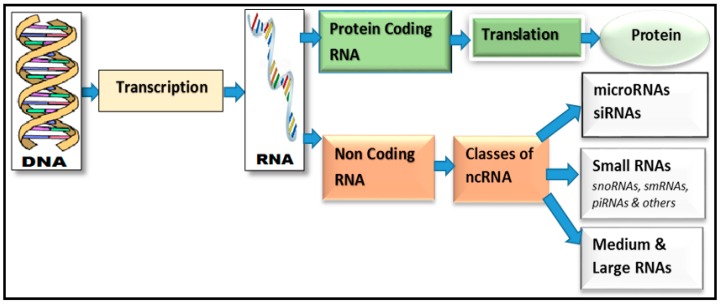
An example of the transcription process to produce protein with coding and non-coding RNA genes.

Non-coding RNAs (ncRNAs) are a type of RNA [1] that is unable to produce a protein. However, these ncRNAs contain unique information that yields other functional RNA molecules [2], and, thereafter, these RNA molecules turn into proteins through gene transcription. This process is visually represented in Figure 1 which shows the transcription step of ncRNA genes. In physiology and disease development, these RNAs regulate numerous levels of gene expression. The current study of the human genome [3] yielded many regulatory ncRNAs including microRNAs, small RNAs, and various types of long ncRNAs (lncRNAs) [4]. In practice, ncRNAs also achieve regularity through modularity, assembling diverse combinations of proteins and possibly RNA and DNA interactions [5]. A regulatory framework was proposed in [6] to construct a network between long ncRNAs (lncRNAs) and protein-coding genes using the Bayesian network (BN). They utilized 762 prostate RNA-seq data to construct this regularity network. In that system, it was noticed that the lncRNAs are utilized in tissue development. Apart from the functions of ncRNAs sequences, the ncRNAs are also described by specific secondary and tertiary structures [7]. Still, finding the function and structure of ncRNAs is becoming a challenging task due to huge volumes of data involved in human next-generation sequencing (NGS) [8]. For determining the function or annotation of ncRNAs, the computational intelligence (CI) techniques were developed in the past studies. Currently, the CI techniques applied on large human NGS datasets are a challenging task. Despite this fact, the prediction of ncRNAs [9] is also a major issue for CI techniques. 

We present a detailed review on state-of-the-art computational intelligence (CI) techniques from 2001 to 2016 in terms of automatic functional annotation and finding of non-coding RNA (ncRNAs) genes. The primary aim of this review article is to attract both biologists and computer experts to show the problems and importance of CI techniques for the finding of human disease in various domains. In the literature, researchers are mainly using CI algorithms such as support vector machine (SVM), neural network (NN), Bayesian networks (BNs), genetic algorithms (GAs), hidden Markov models (HMMs), and hybrid classifiers to find ncRNA genes. The latest trend is to develop more advanced CI techniques that will try to classify or annotate the ncRNA genes. Currently, the authors are widely using deep neural network (DNN) learning and/or convolutional neural network (CNN) classifiers to predict the ncRNAs sequences. The DNN is a more advanced CI technique to recognize multiclass specific problems without using domain-expert knowledge.

However, there are a number of challenges to annotating ncRNAs [10] because there are many classes that are predicted by medical and bioinformatics experts. This happened due to a lack of an unambiguous classification framework in past studies. Similarly, the differentiation [11] between lncRNAs and messenger RNAs (mRNAs) was also a challenging task. Compared to lncRNA patterns, the microRNA (miRNA) [12] is a type of ncRNA that regulates the gene expression during post-transcriptional operation. It was noticed that microRNA has some special roles in the development of cancer cells. However, the review suggests that the functional identification of miRNAs continues to be a thought-provoking task due to more than 1000 distinct genes of miRNAs in the human genome. Furthermore, we also describe data sources that are provided to facilitate the researchers in the development of computational algorithms. This review will serve as a good reference to the newcomers in the computational domain field of ncRNA research. Moreover, this review article focuses on the technical site and the limitations of state-of-the-art CI techniques.

This review article is organized as follows: Section 2 describes the detailed state-of-the-art computational intelligence (CI) approaches for finding ncRNAs and miRNA genes. The online tools and data sources are also presented for the researchers to analyze ncRNA genes. The discussions are presented in Section 3 and conclusions and future works are presented in Section 4 and Section 5, respectively.

## 2. Review of Computational Intelligence Techniques

There has been no clear review article about CI techniques for finding ncRNA and miRNA genes, according to our knowledge, that focuses on CI methods from 2001 to 2016. This methodological review about CI techniques is presented in Table 1 and summarized in Table 2. The primary aim of this review article is to attract both computer and bioinformatics researchers and to make a key reference for further study. In the literature from 2001 to 2016, the CI techniques are developed for finding ncRNA and miRNA genes by using neural networks (NNs), support vector machines (SVMs), Bayesian Networks (BNs), Hidden Markov model (HMM), genetic algorithm (GA), and deep learning. These computational approaches are described in detail in the following sub-sections. The identification of ncRNA [5] functions are an emerging trend in the investigation of human diseases [2] such as cancer, neurological, or cardiovascular disorders. As a result, there has been an increasing interest in the prediction of ncRNA genes.

### 2.1. Predicting Non-Coding RNAs

The studies that used early systematic approaches did not focus on computational methods to predict the function of RNA genes. In [13], the authors developed a CI approach for identification of functional RNA genes. They used SVM and NN for prediction of RNA genes. They achieved 80%–90% classification accuracy using the NN approach and 90%–99% classification accuracy using the SVM classifier. Afterwards, the authors developed many systems for prediction of ncRNA by utilizing the computational algorithms.

Whereas in [14], the SVM algorithm was implemented in graphics processing units (GPUs) based parallel technology to classify ncRNA genes. Large-scale genomic sequence data was utilized to detect these ncRNA sequences. In fact, the authors reported that the detection rate of ncRNA genome sequences are fast using GPU and parallel based hardware implementation.

By using data mining algorithms, a new web-based interface was developed in [15] to detect ncRNAs that are not transcribed into proteins. They named this project “ncRNAclass” (https://biotools.ceid.upatras.gr), which can select efficient features and describe effectively the class of ncRNAs compared to other online tools. The ncRNA class tool is used to differentiate between well-known classes and to target predicted classes of mRNA.

Wang et al. [16] developed a positive sample only learning algorithm to identify ncRNA. Due to a lack of appropriate negative training samples they developed a positive sample-only learning algorithm to identify non-coding and coding RNAs. Using a supervised machine learning SVM, the authors classified transcripts according to their features.

Liu et al. 2006 [17] introduced a coding or non-coding (CONC) method to differentiate non-coding and coding RNAs. They trained a SVM on eukaryotic ncRNA from RNAdb [18] and NONCODE [19] databases. The SVM predicted that coding RNAs were 97% and non-coding were 94%, where means of F-measures were obtained from cross-validation and with a range of 96.66%–98.2% and a standard deviation of 0.6.

The motivation of this study was from the above mentioned CONC method proposed by Lei et al., named [20], coding potential calculator (CPC), which employed a SVM to identify ncRNA using six features that have meaningful biological sequence extracts from the transcript’s nucleotide sequence. The dataset used Rfam [21] and RNAdb [18] for noncoding and European Molecular Biology Laboratory for coding sequence (EMBL CDS) [22].

Sætrom et al. [23] developed a boosted genetic programming method to automatically discover a sequence pattern to predict ncRNAs. The main advantages of this method are that it can use the DNA sequence directly as input, works well with larger sequences, is robust with noise training data, can predict ncRNAs, and does not rely on sequence conservation. On the other hand, Sætrom et al. [24] provided an overview of context sensitive Hidden Markov Models (csHMMs) to predict ncRNA genes. The csHMMs can serve as an efficient framework for these purposes; they also provided an overview of the role of csHMMs in the RNA secondary structure analysis and the prediction of ncRNA genes.

Scott et al. [33] proposed a GA as an alternative to programming for searching ncRNAs. The authors also used decision trees generated by GAs. Scott et al. [34] suggested a covariance model with a different approach in which the space of the starting positions, sequence lengths, and insertion/deletion patterns are searched using a GA. Yan-ling Y. et al. [32] used the Z-curve method for the analysis and search of ncRNAs. DNA sequence can be uniquely represented in 3D space using Z-curves. From the NONCODE [19] database, fifteen disease related ncRNAs sequences were selected for mapping and comparison. The ncRNA sequences in the cellular processes and the base content in these sequences have almost the same Z-curves, even though they are coming from different organisms.

Rivas and Eddy [40] implement a comparative sequence analysis algorithm that tests the patterns of substitutions in pairs of homologous sequence alignments. They developed “pair grammars” based on stochastic models and HMM, whose results compare favorably when tested on known RNAs from BLASTN queries [41]. McCutcheon et al. [37] extended this work with experimental characterization. In addition to having a high false positive rate, QRNA fails to identify ncRNAs without significant structure while also picking up *cis*-regulatory mRNA structures. The results illustrate the importance of having multiple genome sequences at various evolutionary distances available for comparative genomics.

Numata et al. [38] continued this two-tiered “genome based” and “transcripts based” approach to try to identify mammalian mRNA-like processed ncRNAs. They initially eliminated sequences showing homology with known protein sequences and mapped the remainder to the mouse genome before comparing alignments with GENSCAN [42]. This helped them focus on a more manageable 13% of the original set of some 33,000 transcriptional units. Schattner et al. [39] used local base-composition statistics to identify regions of three test genomes that may hold ncRNAs by looking beyond the local percentage of GC bases ((G + C)%) to Chargaff differences to try to eliminate false positives in the filtering process. While reporting significant base-composition variations between RNAs and the background genome, the results may not be applicable to cases with low background (G + C)%. Thao et al. [31] presented an NN-based meta-learner for the de novo method to identify ncRNAs; this method uses sequence and structure based features that are easily derivable from any organism that may is newly or partially sequenced. The main advantage of this method is that it does not require prior homology, multiple sequence alignments, or structural conservation and, thus, can be directly used on any organisms that are newly or partially sequenced.

The significance of ncRNS classes like lncRNA and miRNA is increasing rapidly [43]. Therefore, in this review, we focus on the recently identified novel mechanisms of action, and discuss the current strategies of finding and designating miRNA genes. The development of miRNA and ncRNAs targeted strategies is challenged by several obstacles.

### 2.2. Finding MicroRNAs

In 2005, Chenghai et al. [36] defined a method to classify real and pseudo mRNA by applying SVM using local structure sequence features. They achieved 90% accuracy on human data. Similarly, in [29], a multilayer ANN classifier was proposed by training 17 parameters to predict a real pre-miRNA or a pseudo pre-miRNA. On average, a sensitivity of 97.40% and a specificity of 95.85% were obtained. This approach was also compared with another four state-of-the-art classification methods: MiPred [44], MiPred [45], miRabela [46], microPred [47], and Triplet-SVM [36] classifier. A new classifier was developed in [30] to predict the regulation of miRNA. In that study, they showed that the state-of-the-art methods are adequate for determining the pre-miRNA. However, the authors developed a system to improve the precision performance of pre-miRNA that can handle a new multiple-stem and loop-secondary structure features by using neural networks. The real pre-miRNA dataset was utilized to successfully construct this new classifier to manage these class imbalance problems. The 5-fold cross-validation method was also used to evaluate the performance of the proposed classifier. In [48] authors have used a hybrid approach to predict small noncoding RNAs genes. In [29], a supervised NN machine learning approach was developed to predict new miRNA known as pre-miRNAs on a set of coding sequence (CDS) human regions. In that research, the obtained results (99.9% of accuracy (ACC), 99.8% of sensitivity (SN), and 100% of specificity (SP)) provided a more reliable prediction. The experimental results indicated that the miRNA achieves better results than other approaches and declares it to be the most effective tool to predict novel miRNAs. A miRNA target prediction algorithm was proposed in [27] by contrast relaxing and CNN methods. In that study, the input dataset was artificially generated by CNN for the prediction of the target of miRNA when this mechanism is poorly known. To avoid inaccurate prediction, they used the contrast relaxing method to construct a balanced training dataset. The obtained results indicate that they achieved higher values of SN of 88%, SP of 94%, and ACC of 90%.

In [25], the classification of miRNAs was proposed to differentiate between normal and tumor tissues by using a multi-objective evolutionary-optimization technique. In that optimization strategy, the automatic selection of the classifier, its parameters, and feature combination steps were performed. This approach was divided into two steps. Firstly, they used a multi-objective algorithm with four classifiers such as random tree (RT), random forest (RF), sequential minimal optimization (SMO), and logistic regression. Afterwards, the multi-objective algorithm was automatically determined using the classifier, its parameters, and feature sets. In that study, the authors implemented a multi-objective evolutionary method to examine the search capability of non-dominated sorting genetic algorithm (NSGA)-II. The obtained results were also compared with several state-of-the-art methods on mRNA and miRNA datasets. An automatic miRNAs target prediction (deepTarget) algorithm was developed in [26] by using the deep NN based approach to reduce manual selection of features. They showed that many computational tools have been developed to solve this problem, but the false positive rate was high. In that study, the performance of the deepTarget algorithm delivered more than a 25% increase in the F-measure compared to that of the state-of-the-art target prediction algorithms.

## 3. Online Tools and Data Sources

The online tools and data sources are provided for researchers to develop new studies based on new CI approaches. We did not only show the sources for finding ncRNA and microRNA genes, but also other classes of non-coding RNAs—as displayed in Table 3. Whereas in Table 1, the online available tools were described. As mentioned before, these databases are useful to test various annotation and gene-finding techniques. The Rfam database [21] was one of the first. It integrated various new and existing curated structural alignments into a common structure-annotated format. It also uses covariance modeling and automated sequence annotation software.

The NONCODE database [19] brought together most publicly available information about experimentally confirmed or computationally predicted ncRNAs with the exception of transfer RNAs (tRNAs) and ribosomal RNAs (rRNAs). It also introduced a classification system termed process function class (PfClass) based on the cellular processes and functions associated with the ncRNA.

The RNAdb [18] was launched as a sequence repository for experimentally supported regulatory mammalian ncRNAs (miRNAs, small nucleolar RNAs (snoRNAs), but not tRNAs, rRNAs, and spliceosomal RNAs). Apart from bioinformatics analyses, it also was meant to facilitate microarray chip characterization experiments. This database also includes a large number of commonly accepted ncRNAs from reputable complementary DNA (cDNA) libraries. The authors described the computational methods to identify genes and presented a brief technical reference for future studies.

One of the first programs for searching a sequence database for homologs of a single RNA molecule on the basis of secondary structure was RSEARCH [49]. It relies on a local alignment algorithm. The latter are a series of base pair and single nucleotide substitution matrices for RNA sequences. The web-based tool RNALOSS [9] was developed to provide information about the distribution of locally optimal secondary structures.

FastR [51] was applied to the discovery of riboswitches, a class of RNA domains, which regulate metabolite synthesis. Given an RNA sequence with known secondary structure, FastR efficiently computes all structural homologs in a genomic database. The tool relies heavily on filter design and optimization as well as the actual filtering algorithms and computation.

## 4. Discussion

The interpretation and annotation of ncRNA gene finding is an emerging trend. There are numerous challenges to annotate and interpret ncRNAs because there are many classes that are still being predicted by medical and bioinformatics experts [52,53]. In recent years, the CI approaches have attracted many researchers to perform those tasks. However, those CI approaches were lacking a definitive classification framework that utilized the past studies. Some reviews have summarized CI approaches but focused on the particular viewpoint on methodologies. In this article, the CI techniques for interpretation and annotation of ncRNA gene finding are summarized in detail differently from the existing body of research, and we attempt to deliver a short but concise technical discussion.

Biological sequences (such as DNA, RNA, and protein sequences) naturally fit the recurrent NN that are capable of temporal modeling. Nonetheless, prior work on applying deep learning to bioinformatics utilized only convolutional and fully connected NNs. The biggest novelty of our work lies in its use of recurrent NNs to model RNA sequences and to further learn their sequence-to-sequence interactions, without laborious feature engineering (e.g., more than 151 features of miRNA-target pairs have been proposed in the literature). As shown in their experimental results, even without any of the known features, deepTarget delivered substantial performance boosts (over 25% increase in F-measure) over existing miRNA target detectors, demonstrating the effectiveness of recent advances in end-to-end learning methodologies.

The training deepTarget [26] was focused on improving its capability to reject false positives (i.e., bogus miRNA-mRNApairs) as a target predictor. The decision was based on the study that more priority should be given to sensitivity in the search for potential targets of specific miRNAs, whereas specificity should be emphasized in the examination of miRNAs that regulate specific genes. Depending on the specific needs, we could alternatively train deepTarget to put more priority on specificity. For instance, this could be done by altering the composition of a mock negative dataset to have additional mispairings between miRNA and mRNA sequences except the seed sequence.

Notably, deepTarget [26] does not depend on any sequence alignment operation, which has been used in many bioinformatics pipelines as a holy grail to reveal similarity/interactions between sequences. Although effective in general, sequence alignment is susceptible to changes in parameters (e.g., gap/mismatch penalty and match premium) and often fails to reveal the true interactions between sequences, as is often observed in most of the alignment-based miRNA target detectors. By processing miRNA and RNA sequences with recurrent neural networks (RNN)-based auto encoders without alignment, deepTarget successfully discovered the inherent sequence representations, which are effectively used in the next step of deepTarget for interaction learning. Although the performance of deepTarget is incomparably higher than that of the existing tools we compared it to, Fritah et al. [54] there remains room for further improvements. An additional breakthrough may be possible by enhancing the current step to learn sequence-to-sequence interactions. The current version of deepTarget relies on concatenating the RNA representations from two auto-encoders and learning interactions therein using a unidirectional two-layer RNN architecture. Although this architecture was effective to some extent, as shown in their experiments [26], adopting even more sophisticated approaches may further boost the capability of deepTarget to detect subtle interactions that currently go undetected. 

As we are living in the era of big data, transforming biomedical big data [1] into valuable knowledge has been one of the most important problems in bioinformatics. At the same time, deep learning has advanced rapidly since early 2000s and has recently shown state-of-the-art performance in various fields. This article reviews some research of deep learning [55] in bioinformatics. To provide a big picture the authors in the past studies utilized deep learning architectures (i.e., deep neural network, convolutional neural network, recurrent neural network, modified neural network) and presented brief descriptions of each work. Additionally, we introduce a few issues of deep learning in bioinformatics such as problems of class imbalance data and suggest future research directions [28] such as multimodal deep learning. The authors believe that the study could provide valuable insights and be a starting point for researchers to apply deep learning in their bioinformatics studies.

Certainly, bioinformatics is no exception in such trends. Various forms of biomedical data including omics data, image, and signal have been significantly accumulated, and its great potential in biological and health-care research has caught the interest of industry as well as academia. For instance, IBM provided Watson for Oncology, a platform analyzing patients’ medical information and assisting clinicians with treatment options [2,3]. In addition, Google DeepMind, achieving a great success with AlphaGo in the game of GO, recently launched DeepMind Health to develop effective healthcare technologies [4,5].

To extract knowledge from huge data in bioinformatics, machine learning has been one of the most widely used methodologies. Machine learning algorithms use training data to uncover underlying patterns, build a model, and then make predictions on the new data based on the model. Some of the well-known algorithms—SVM, HMM, BNs, Gaussian networks—have been applied in genomics, proteomics, systems biology, and many other domains [7,55]. Conventional machine learning algorithms have limitations in processing the raw form of data, so researchers put a tremendous effort into transforming the raw form into suitable high abstraction level features with considerable domain expertise [56]. On the other hand, deep learning, a new type of machine learning algorithm, has emerged recently on the basis of big data, the power of parallel and distributed computing, and sophisticated algorithms. Deep learning algorithms have overcome the former limitations and are making major advances in diverse fields such as image recognition, speech recognition, and natural language processing.

Certainly, bioinformatics is no exception in deep learning applications. Several studies have been conducted [57] to apply deep learning in bioinformatics as in Figure 1. We categorized the research by the form of input data into three domains: omics, biomedical imaging, and biomedical signal processing. Detailed lists of bioinformatics research topics where deep learning is applied and input data examples of each domain are shown in Table 1.

**Figure 2:**
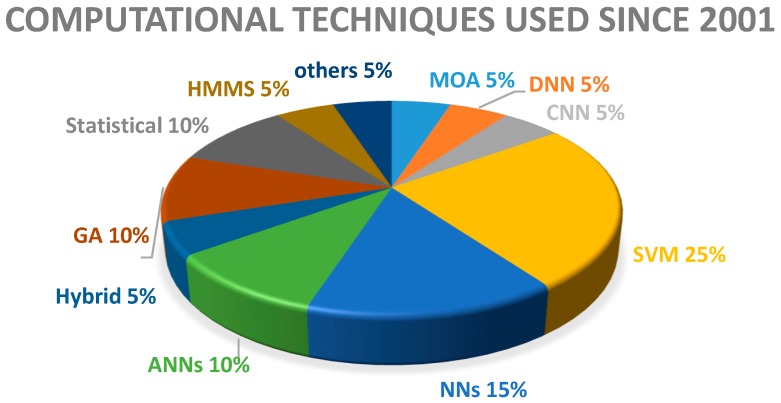
Computational techniques (percentage) used since 2001. MOA: Massive online analysis, DNN: Deep neural network, CNN: Convolutional neural network, SVM: Support vector machine, NNs: Neural networks, ANN: Artificial neural Networks, GA: Genetic algorithm, HMMs: Hidden Markov Model.

Figure 2 shows the classification of CI techniques percentage used since 2001. This pie chart clearly shows that most of the CI techniques have been used for this problem. It clearly shows that most of the methods are based upon ANN such as CNN and DNN that is approximately 35% of all these CI techniques. It clearly shows that ANN based approaches are the most promising as compared to other CI techniques. Table 1 shows that the NN based approach achieved approximately 99% accuracy to predict new miRNA known as pre-miRNAs [28] which is the maximum so far. Mostly NN based approaches have been proposed recently. Figure 2 also clearly shows that SVM has also been used for this problem around 25% of the time. But Table 1 shows that the maximum accuracy achieved by the SVM based approach is 95%, which is less than that of the NN based approaches. SVM based approaches were proposed before 2010 as shown in Table 1. Table 1 also shows that some other classifiers such as RT, RF, SMO, HMM, GA, and logistic regression have also been used, but these approaches do not show promising results as compared to NN based approaches. Thus, it can be concluded that NN based approaches are the most suitable for the classification and prediction of miRNA.

In fact, the differentiation between normal and cancer tissues are dependent on the analysis of the lncRNA transcription patterns. It was also noticed that the lncRNA expression in normal tissues is highly abnormal for lncRNA expression in human cancers. Therefore, they utilized 272 human serial analyses of gene expression (SAGE) libraries to detect transcription patterns of lncRNA [58].

State-of-the-art advances have been presented in three levels of lncRNAs (the primary sequence, the secondary structure, and the function annotation) along with CI methods [59]. Computational approaches for the analysis of ncRNA through deep sequencing techniques were discussed in [60]. One review of lncRNAs [23] also argues that the quality of annotations and the function of these genes are important. In that research study, the authors proposed a novel cancer-related finding of the lncRNAs gene and discussed the limitations.

## 5. Conclusions

This paper comprehensively reviews the state-of-the-art CI techniques starting from 2001 to up till now in terms of automatic functional annotation and finding ncRNAs human genes. It concluded that the past CI approaches lacked a definitive classification framework and focused on a specific usage of machine learning algorithms without doing methodological contribution. In order to functionally annotate ncRNAs and find mRNAs, the researchers are widely using machine learning algorithms such SVM, NN, BNs, GAs, and HMMs. In practice, these conventional machine learning algorithms require domain-expert knowledge for pre-processing of raw input data and the selection of features and to fine-tune some parameters for increasing the accuracy. A lot of effort is required for pre- and post-processing to achieve the up-to-the-mark optimization results. Alternatively, a new machine learning concept known as deep neural network (DNN) [26] has emerged recently to control the problem of pre- and post-processing steps and domain-expert knowledge to select the features from huge raw data which is especially required in the case of human genome sequencing. Most recently, there were some studies [26,27] that focused on deep learning algorithms for the prediction of lncRNAs. Definitely, the deep learning machine learning algorithms is a latest trend that did not require pre- and post-processing classification steps to handle the big raw human genomics data. In brief, the up-coming advances in the methodological formation of deep machine learning algorithms for ncRNAs can provide excellent performance to further investigate functional annotation and to find mRNAs in the future.

## Figures and Tables

**Table 1:** State-of-the-art computational intelligence (CI) techniques for finding non-coding RNA (ncRNA) genes from 2001 to 2016.

Cited	Approach	^4^ CMP	Results (%)	Methodology and Online Resource Tools
[13]	A computational approach to identify genes for functional RNAs in genomic sequences.	√	^2^ S: 90%, ^3^ P: 99%	NN and SVM. Online tool unavailable.
[14]	To detect ncRNA sequences.	×	^−−−−−−^	The support vector machine (SVM) algorithm was implemented in graphical processing units (GPUs) based parallel technology. Online tool unavailable.
[15]	To differentiate between well-known classes and target predicted classes of messenger RNA (mRNA).	√	^−−−−−−^	A new web-based interface was developed to detect ncRNAs. Available at http://biotools.ceid.upatras.gr/ncrnaclass/.
[16]	To identify ncRNA a positive sample only learning algorithm is introduced.	×	^1^ A: 80%	The SVM used as the core learning machine assessed by 5-fold-validation in recovery of known ncRNA. Data available online at (http://bioinformatics.oxfordjournals.org/content/22/21/2590/suppl/DC1)
[17]	To introduce a method to differentiate between coding or non-coding RNA.	×	^3^ P: 97%, ^2^ S: 98%	Supervised machine learning SVM is used to classify transcripts according to features they would have if transcripts coded for proteins. Online data source of mRNA at: RNAdb (http://research.imb.uq.edu.au/rnadb).
[20]	To identify ncRNA using six features extracted from transcript’s nucleotide sequence.	×	^−−−−−−^	SVM (coding potential calculator ((CPC)) to identify ncRNA using six features extracted from transcript’s nucleotide sequence. Dataset used Rfam and RNAdb for noncoding and EMBL CDS for coding. Online web-based interface available of CPC at http://cpc.cbi.pku.edu.cn.
[23]	The prediction of ncRNA genes using boosted genetic programming.	×	^1^ A: 80%	The GA and 10-fold cross validation was used to train and test the learning machine. Online tool unavailable.
[25]	To classify micro RNAs (miRNAs) and to differentiate between normal and tumor tissues.	√	^−−−−−−^	A multi-objective algorithm was developed by using four classifiers such as random tree (RT), random forest (RF), sequential minimal optimization (SMO) and logistic regression (LR).
[26]	To automatically predict miRNA target.	√	F-measure: 0.95	The deep neural-network (DNN) was utilized to increase F-measure by 25% for prediction of miRNA targets. Available at (http://data.snu.ac.kr/pub/deepTarget)
[27]	To predict miRNAs targets.	×	^1^ A: 90%, ^2^ S: 88%, ^3^ P: 94%	Contrast relaxing and convolutional neural network (CNN) methods. Online tool unavailable.
[28]	To predict new miRNA, known as pre-miRNAs.	×	^1^ A: 99.9%, ^2^ S: 99.8%, ^3^ P: 100%	A neural networks (NNs) classifier was used to predict miRNA. Online tool unavailable.
[29]	To improve the performance and to predict the regulation of miRNA.	×	^−−−−−−−−^	The authors utilized a NNs classifier to predict miRNA. Online tool unavailable.
[30]	To predict a real pre-miRNA or a pseudo pre-miRNA.	√	^1^ S: 97.40%, ^2^ P: 95.85%	The authors utilized a multilayer artificial neural network (ANN) classifier. Online tool unavailable.
[31]	A de novo prediction algorithm to identify ncRNA using features derived from sequence and structure of known ncRNA.	×	^2^ S: 68%, ^3^ P: 70%, ^1^ A: 70%	NN-based meta-learner de novo predictor using folding, ensemble, and structure-based features. Online data and program found at: http://csbl.bmb.uga.edu/publications/materials/tran/
[32]	The 15 disease related ncRNAs sequences are utilized from the ncRNAs with Alzheimer disease.	×	^−−−−−−^	From the NONCODE database [19], 15 disease related ncRNA sequences were selected for mapping and comparison. The ncRNA sequences in the cellular process and the base content in these sequences have almost the same Z-curves even though they are coming from different organisms. Online tool unavailable.
[33]	To identify ncRNA genes using a genetic algorithm (GA).	×	^−−−−−−^	The observed sequence in real sequence data is used to motivate the use of GAs to quickly reject regions of the search space of ncRNAs. Online tool unavailable.
[34]	To identify ncRNA using covariance searching.	×	^−−−−−−^	The covariance models for ncRNA gene finding is extremely powerful and also extremely computationally demanding. Online tool unavailable.
[35]	A comparative genomic approach is used to detect ncRNA.	×	^−−−−−−^	Developed an efficient clustering method for finding potential ncRNAs in bacteria by clustering genomic sequences. Online tool unavailable.
[36]	To identify real and pseudo miRNA using SVM with features that are present in local structure-sequence.	×	^1^ A: 90%	A method to classify real and pseudo miRNA by applying SVM using local structure sequence features. Online tool unavailable.
[37]	Computational identification of ncRNAs in *Saccharomyces cerevisiae* by comparative genomics.	×	^−−−−−−^	Computational screen followed by Northern blot and transcript sequencing. Online tool unavailable. Data set is available only at: http://genome.cshlp.org/content/13/6b/1301/suppl/DC1.
[38]	Identification of putative noncoding RNAs among the RIKEN mouse full-length cDNA collection.	×	^−−−−−−^	The authors identified nine ncRNAs. Online tool unavailable. Data set is available only at: http://genome.cshlp.org/content/13/6b/1301/suppl/DC1.
[39]	The 19 candidate ncRNAs were identified including one with significant homology.	×	^−−−−−−^	The author used base-composition statistics method to find variety of ncRNAs. Online tool unavailable.
[40]	ncRNA gene detection using comparative sequence analysis.	√	^2^ S: 97.3%, ^3^ P: 100%	Comparative sequence analysis algorithm with “pair grammars” based on stochastic and hidden Markov models (HMM). Online tool unavailable.

^1^ A: Accuracy, ^2^ S: Sensitivity, ^3^ P: Specificity, and ^4^ CMP: Comparisons, √: Compared and ×: Not compared.

**Table 2:** A brief summary of CI techniques with respect to classification algorithms.

Year	Computational Intelligence
2016	Multi classifiers (RT, RF, SMO) and Logic Regression LRDNN
2015	CNN, SVM, NN
2012	NN
2009	ANN, De novo NN, Hybrid Methods (HMs)
2008	Z-curve, GA
2007	SVM-Coding
2006	SVM and Covariance model parameter estimation
2005	GA, SVM, HMMs
2002	Local base-composition statistics
2001	Single-hidden layer NNs and SVMs, Comparative sequence analysis algorithm based on HMMs

**Table 3:** State-of-the-art CI online databases for the development of CI techniques.

Cited	Databases	Web-Links
[9]	RNALOSS	http://clavius.bc.edu/~clotelab/RNALOSS
[18]	RNAdb	http://research.imb.uq.edu.au/RNAdb
[19]	NONCODE	http://noncode.bioinfo.org.cn
[21]	Rfam	http://www.sanger.ac.uk/science/tools/rfam
[49]	RSEARCH	http://www.yeastgenome.org
		ftp://ftp.tigr.org/pub/data/a_thaliana/ath1/SEQUENCES
[50]	EICO	http://fantom2.gsc.riken.jp/EICODB/
